# Accelerating the Uptake of WHO Recommendations for Mass Drug Administration Using Ivermectin, Diethylcarbamazine, and Albendazole

**DOI:** 10.4269/ajtmh.21-0972

**Published:** 2022-03-15

**Authors:** Jonathan D. King, Julie Jacobson, Alison Krentel

**Affiliations:** ^1^ World Health Organization, Geneva, Switzerland;; ^2^ Bridges to Development, Seattle, Washington;; ^3^ School of Epidemiology and Public Health, University of Ottawa, Ottawa, Canada

## Abstract

Triple therapy with ivermectin, diethylcarbamazine, and albendazole (IDA) for the elimination of lymphatic filariasis (LF) represents a compelling example of accelerating the timeline from development to introduction and impact. Previous articles outlined how the clinical development process was able to compress timelines and provide the evidence needed for the WHO to issue guidelines on the use of IDA for mass drug administration for LF. We explored the drivers for the rapid and successful introduction of IDA in the early-adopter countries. Lessons from this experience highlight five key elements for moving from WHO recommendations to program uptake after the publication of the guideline: 1) early engagement with stakeholders to create partnerships to coordinate and plan for implementation; 2) recognition by countries and partners of the potential of IDA to improve efforts to eliminate LF; 3) high-level commitment and coordination at regional levels and, most importantly, at the country level; 4) understanding of the perspectives among people living in LF-endemic communities where mass drug administration is warranted; and 5) affirmation of the feasibility of IDA through sharing lessons learned.

## INTRODUCTION

In October 2017, the WHO produced a guideline on alternative mass drug administration (MDA) regimens targeting the neglected tropical disease lymphatic filariasis (LF) that included a recommendation for the use of triple-therapy MDA with ivermectin, diethylcarbamazine, and albendazole (IDA) ^1^—a newly discovered combination of existing medicines. Between publishing the guideline in October 2017 and a global meeting in July 2019 (Global Review of Initial Use of Triple-Therapy Mass Drug Administration and Planning for Accelerated Elimination of Lymphatic Filariasis, Bangkok, Thailand), eight countries (Samoa, American Samoa, Kenya, India, Papua New Guinea, Timor Leste, Malaysia, and Sao Tome and Principe) adopted IDA as an MDA regimen in national neglected tropical disease (NTD) programs (Table 1).

**Table 1:** First countries implementing IDA

2018	2019
American Samoa	Egypt
Fiji	Guyana
Kenya	Malaysia
India	Sao Tome and Principe
Papua New Guinea	Timor Leste
Samoa	

During this period, the WHO supported multinational and national consultations to guide countries on the implementation of the 2017 guidelines that helped move IDA, within 2 years, from WHO policy to national policy to program implementation. Reflecting on the early experiences of IDA implementation, there were several important factors that contributed to the quick and successful transition of global policy to country action, but five key elements were required: 1) early engagement with stakeholders to create partnerships to coordinate and plan for implementation; 2) recognition by countries and partners of the potential of IDA to improve efforts to eliminate LF; 3) high-level commitment and coordination at regional levels and, most importantly, at the country level; 4) understanding of the drivers of acceptance of IDA among people living in communities where the regimen is warranted according to WHO recommendations; and 5) affirmation of the feasibility of IDA by sharing lessons learned from the first countries to use IDA.

## STAKEHOLDER ENGAGEMENT AND COORDINATION

Early planning and partner coordination were key to the successful introduction of IDA. The WHO planned a series of workshops inviting eligible countries, their partners, and global experts to familiarize countries with IDA and to begin planning for how IDA could be used in their local context to accelerate LF elimination. Preparatory work for these meetings began early in anticipation of the release of the new guideline to ensure uptake could start quickly. The initial planning and peer-to-peer sharing through these meetings between countries and partners was an essential element to getting well-thought-out strategic plans for IDA use.

Country programs established national task forces comprised of representatives of national experts in NTDs, pharmacovigilance, primary health care, community groups, and government officials. These task forces met frequently and provided a mechanism to move forward the planning and coordination for IDA introduction in each country. The meetings allowed discussion of the guideline and an open forum to address questions and concerns from all stakeholders. The task forces reviewed the evidence upon which the recommendations were based, including the data on the efficacy, safety, and acceptability of IDA from the field trials. ^2^^–^ ^5^ Importantly, the meetings provided an opportunity for the program to be open to new information and emerging research that could help guide the use of IDA at the national level, and to work collaboratively with partners and other departments to support rollout.

## COUNTRY AND PARTNER WILLINGNESS

Countries first adopting IDA viewed the novelty of the regimen as a potential to bring vigor into established programs and, in some settings, to revitalize aging routine programs. The greater efficacy of IDA meant a potential for stopping transmission with fewer MDA rounds. The extended benefits of ivermectin against other helminths and ectoparasites was also motivating. We learned through the experiences of the early-adopter countries and early qualitative studies that simply introducing the height-based dosing poles in some locations represented something new and interesting in the community, particularly in those areas where MDA for LF had previously used age-based dosing tables. Programs were hopeful that these new aspects and increased interest would result in greater coverage.

Through the meetings and consultations, program staff and health officials discussed the willingness to adopt IDA, but also concerns, and planned for how to address issues as they arose. For example, in American Samoa, as part of the United States, a concern about regulatory approval of IDA was raised that was addressed by applying for and receiving Investigational New Drug approval from the U.S. Food and Drug Administration. Each country raised questions about the potential impact of fear of the number of tablets or of adverse events. This presented an opportunity to establish or improve collaboration between NTD programs and national pharmacovigilance agencies to ensure communities of the availability of care for and close monitoring of adverse events. Presenting data from the safety trial and the acceptability study reassured stakeholders and programs about community response to IDA and levels of adverse events. ^1^^,^ ^2^ Early-adopter countries used novel techniques such as telephone hot lines and Facebook pages to provide community members with access to health personnel during the MDA.

## HIGH-LEVEL COMMITMENT AND COORDINATION

Obtaining support from high-level leadership proved crucial to the successful introduction of IDA in early-adopter countries. Coordination on the introduction and implementation of IDA occurred at the national and regional levels, involving multiple sectors of government, including high-level officials and ministers. The South East Asia Region of the WHO sponsored a regional ministerial meeting (Keeping the Promise: Ending NTDs on Time in the SEA Region, April 2017) to sensitize ministers of new strategies to accelerate elimination of NTDs including IDA. ^6^

Recommendations from the national task forces were submitted to the ministers of health facilitating the official adoption of IDA into national LF elimination programs. Involving ministers and public health officials in operational decision-making and requesting their participation in public launching events raised the visibility of the LF elimination program. ^7^ The first countries using IDA were successful in garnering support of local politicians in IDA MDA launching events. This was important to demonstrate to the community that celebrities and leaders can also have their height measured and take all the tablets safely. For those countries with prior rounds of MDA, this demonstration of renewed support for LF elimination across stakeholders and partners helped signal to community members the importance of MDA participation.

## UNDERSTANDING PERSPECTIVES OF THE COMMUNITY BY INCORPORATING SOCIAL RESEARCH INTO PLANNING

During the community safety studies and the first implementation units where IDA was used in national LF elimination programs, social research was conducted concurrently, providing opportunities to expand understanding on its use. Community acceptability studies yielded important insights and helped inform how IDA would be perceived by communities. ^3^ These studies helped to identify where there had been challenges with MDA reach in the past, and how new messaging and tailored approaches to MDA may yield better coverage with IDA. Specifically, this included community feedback on the number of pills involved in IDA, the perception of adverse events, and any additional benefits of ivermectin. Efforts to coordinate operational research, specifically around acceptance of IDA, allowed results from initial studies to be used to help subsequent programs plan their social mobilization and messaging.

## SHARING EARLY LESSONS LEARNED

Multinational meetings in 2019 provided a space to share documented experiences among early-adopter countries, build partnerships, and identify best practices across countries and regions. Case studies from the first program implementation experiences presented at these meetings helped expand understanding of IDA use outside the research trial setting. This process generated confidence in the feasibility of implementation, affirmed the safety and acceptability of the new MDA regimen, and informed planning for potential next-wave adopters. During these meetings, countries presented their new or modified approaches to community engagement and social mobilization. Programs reported on the methods of distribution used, dosing strategy (height, weight, or age), and monitoring of directly observed therapy such as finger-marking with indelible ink. Countries that had not yet started IDA were interested in the additional, improved efforts invested in monitoring for adverse events. Introducing microplanning as a tool to improve MDA was discussed as well as new aspects of drug distributor training and innovative interpersonal communication. For programs considering the introduction of IDA, hearing from the experiences of their peers fostered reassurance as they witnessed practical and creative ways of incorporating IDA successfully.

## CONCLUSION

The transition from WHO policy to integration of IDA into country NTD programs has been successful in a relatively short timeline. By the end of 2019, 11 countries spanning all five LF-endemic regions had implemented IDA, treating more than 45 million people. ^8^ This was possible as a result of five elements: 1) early engagement with stakeholders, 2) the potential of IDA being recognized by countries and partners , 3) high-level commitment and coordination at regional and country levels, 4) inclusion of the end user through the incorporation of social research, and 5) sharing experiences between countries. The onset of the coronavirus pandemic in 2020 has slowed progress in the implementation of IDA. Since the end of 2019, only three additional countries have adopted IDA and only six countries have been able to implement second rounds of IDA MDA in the initial areas covered. ^9^ This article highlights briefly some important shared factors in the successful transition of policy to action at the national level. More in-depth firsthand experience from IDA implementation leaders in a set of early-adopter countries has been documented. ^10^^,^ ^11^
